# Underreporting of acute kidney injury in randomized trials of acute respiratory distress syndrome with mortality endpoints: a systematic review

**DOI:** 10.62675/2965-2774.20260422

**Published:** 2026-07-22

**Authors:** Rogerio da Hora Passos, Bruno Zawadzki, Luis Claudio Santos Pinto, Rafael Hortencio Melo, Thais Dias Midega, Bruno de Arruda Bravim, Vagner Pires de Campos, Arnaldo Alves da Silva, Prashant Nasa, Fernanda Oliveira Coelho

**Affiliations:** 1 DaVita Tratamento Renal Rio de Janeiro RJ Brazil DaVita Tratamento Renal - Rio de Janeiro (RJ), Brazil; 2 Universidade Federal do Pará Department of Nephrology Belém PA Brazil Department of Nephrology, Universidade Federal do Pará - Belém (PA), Brazil; 3 Hospital Israelita Albert Eisntein Departamento de Pacientes Graves São Paulo SP Brazil Departamento de Pacientes Graves, Hospital Israelita Albert Eisntein - São Paulo (SP), Brazil; 4 New Cross Hospital Department of Anaesthesia and Critical Care Medicine The Royal Wolverhampton NHS Trust Wolverhampto United Kingdom Department of Anaesthesia and Critical Care Medicine, New Cross Hospital, The Royal Wolverhampton NHS Trust - Wolverhampton, United Kingdom

**Keywords:** Acute kidney injury, Multiple organ failure, Organ dysfunction scores, Respiratory distress syndrome, Renal replacement therapy, Mortality

## Abstract

**Objective:**

To examine how acute kidney injury is represented and reported in randomized controlled trials of acute respiratory distress syndrome with mortality as the primary endpoint.

**Methods:**

This descriptive systematic review included parallel-arm randomized controlled trials enrolling adult patients with acute respiratory distress syndrome, defined by American-European Consensus Conference or Berlin criteria, in which mortality was the primary outcome. The protocol was prospectively registered in PROSPERO (CRD420251043094). Searches were conducted in PubMed^®^/MEDLINE^®^, Embase, and Scopus for studies published between January 1st, 2005, and April 10, 2025. Two reviewers independently screened studies and extracted data by consensus. Data extraction focused on acute kidney injury reporting, use of standardized definitions, serum creatinine, renal replacement therapy, fluid balance, and kidney-related subgroup analyses.

**Results:**

Twenty-seven randomized controlled trials met inclusion criteria. None applied standardized acute kidney injury definitions such as KDIGO, AKIN, or RIFLE. Serum creatinine was reported in two trials and renal replacement therapy in four, without details on timing or criteria. Organ dysfunction scores were commonly reported, but renal subscores were not specified. Fluid balance was reported in five trials but was not included in the adjusted analyses. No study performed mortality analyses stratified by acute kidney injury or baseline kidney function.

**Conclusion:**

Acute kidney injury is inconsistently represented in randomized trials of acute respiratory distress syndrome evaluating mortality. This inconsistency limits the interpretation of extrapulmonary organ dysfunction. Recognizing the multisystem nature of critical illness may improve the clinical relevance of future trial designs.

## INTRODUCTION

Acute respiratory distress syndrome (ARDS) is a major cause of morbidity and mortality in critically ill patients.^(1)^ Defined by non-cardiogenic pulmonary edema, severe hypoxemia, and reduced lung compliance, ARDS often requires prolonged mechanical ventilation and intensive care.^(2)^ While the syndrome is primarily pulmonary in presentation, its systemic implications are substantial; multiorgan dysfunction is common, and the kidneys are particularly vulnerable.^(3)^ Despite this, the consideration of kidney-related outcomes in randomized trials designed to evaluate mortality in ARDS remains variable.

Acute kidney injury (AKI) occurs frequently in patients with ARDS and is associated with poor outcomes, including longer intensive care unit (ICU) stays, increased need for renal replacement therapy (RRT), and higher mortality.^(4)^ The interplay between lung and kidney injury involves complex hemodynamic, inflammatory, and neurohormonal mechanisms.^(5)^ Clinical management strategies such as fluid therapy, ventilator settings, and pharmacologic interventions can directly influence both pulmonary and renal function.^(6)^ As such, the kidney is not just a bystander in ARDS but a key determinant of disease trajectory.^(7)^ Consistent with this systemic perspective, ARDS is increasingly recognized as a biologically heterogeneous syndrome, with distinct subphenotypes characterized by different inflammatory profiles, degrees of extrapulmonary organ dysfunction, and clinical outcomes. Hyperinflammatory subphenotypes have been associated with a higher incidence of metabolic acidosis and increased creatinine levels, as well as worse clinical outcomes.^(8)^

Recent patient-level analyses from over 5,000 participants in 10 ARDS trials revealed a 43.7% incidence of AKI, with stable risk over time and an estimated 15.4% excess 90-day mortality attributable to AKI rising to 20.3% in severe cases.^(9)^ Most AKI occurred early during critical illness and was linked to factors such as hemodynamic instability, inflammation, and ventilator-associated injury.^(10)^ Despite advances in ICU care, no improvement in AKI incidence or renal recovery was observed, underscoring the need for consistent reporting and kidney-specific endpoints in ARDS trials.^(11)^

Taken together, these observations highlight the potential gap between the systemic nature of ARDS and the outcomes typically reported in trials that shape clinical practice. The aim of this systematic review was therefore to examine how AKI is captured in ARDS RCTs that use mortality as a primary endpoint. Specifically, we sought to determine how often AKI is reported as an outcome, whether standardized definitions are applied, and to what extent trials report RRT use, assess fluid balance, or include kidney-related subgroup analyses.

## METHODS

### Study design

This study is a descriptive systematic review of randomized controlled trials evaluating adult patients with ARDS in which mortality was the primary outcome, with a focus on AKI and related renal variables.

### Review registration and reporting guidelines

The protocol was prospectively registered with PROSPERO on April 29, 2025, and formally registered on June 30, 2025 (PROSPERO registration number: CRD420251043094). Reporting followed the Preferred Reporting Items for Systematic Reviews and Meta-Analyses (PRISMA) guidelines.

### Eligibility criteria

We included parallel-arm randomized controlled trials conducted in adult populations (≥ 18 years) with ARDS, as defined by either the American-European Consensus Conference (AECC) or Berlin criteria, published in English between January 1st, 2005, and April 10, 2025. Only trials reporting mortality as a primary endpoint were eligible. Review articles, editorials, observational studies, and non-randomized interventions were excluded. No restrictions were applied regarding intervention type, comparator, or ventilatory strategy; included trials evaluated any therapeutic or supportive intervention in the context of ARDS.

### Outcomesa

The primary focus was on the representation and reporting of AKI. This included whether AKI was included as a secondary or exploratory outcome, whether formal definitions (such as Kidney Disease: Improving Global Outcomes [KDIGO], Acute Kidney Injury Network [AKIN], or RIFLE [acronym from *R*isk of renal dysfunction, *I*njury to kidney, *F*ailure or *L*oss of kidney function, and *E*nd-stage kidney disease]) were used, whether serum creatinine or RRT was reported, whether AKI was classified as an adverse event, whether subgroup analyses by AKI status were performed, and whether fluid balance was reported in relation to renal function.

### Search strategy, study selection and data extraction

A comprehensive search was conducted in PubMed^®^/MEDLINE^®^, Embase, and Scopus using controlled vocabulary (MeSH) and relevant keywords. The following terms were used: ("Acute Respiratory Distress Syndrome" [Title/Abstract] OR "ARDS" OR "acute lung injury") AND ("Mortality" [Mesh] OR mortality OR "Death") AND ("Randomized Controlled Trial" [Publication Type] OR "Randomized Controlled Trials" [Mesh] OR "randomized trial" [Title/Abstract]). The search included studies published between January 1st, 2005, and April 10, 2025, and was limited to English-language publications. Two reviewers independently screened all titles and abstracts for eligibility. Full-text articles were retrieved for potentially eligible studies and reviewed in detail. Discrepancies were resolved through discussion and consensus between the two reviewers. Data were independently extracted using a standardized data collection form. Extracted variables included study design, setting, intervention type, ARDS severity, COVID-19 status, reporting of AKI and its definitions, serum creatinine monitoring, RRT use, fluid balance reporting, and whether AKI was analyzed as an adverse event or in subgroup analyses.

### Risk of bias assessment

As no meta-analysis was planned and effect sizes were not pooled, formal risk-of-bias tools were not applied. Instead, methodological completeness and the consistency of AKI-related reporting were qualitatively assessed across trials.

### Data synthesist

Findings were synthesized descriptively. Reporting practices regarding AKI were compared across the included studies. Trends in AKI representation over time, by intervention type, and by coronavirus disease 2019 (COVID-19) *versus* non-COVID-19 status were evaluated narratively.

## RESULTS

**Figure 1:**
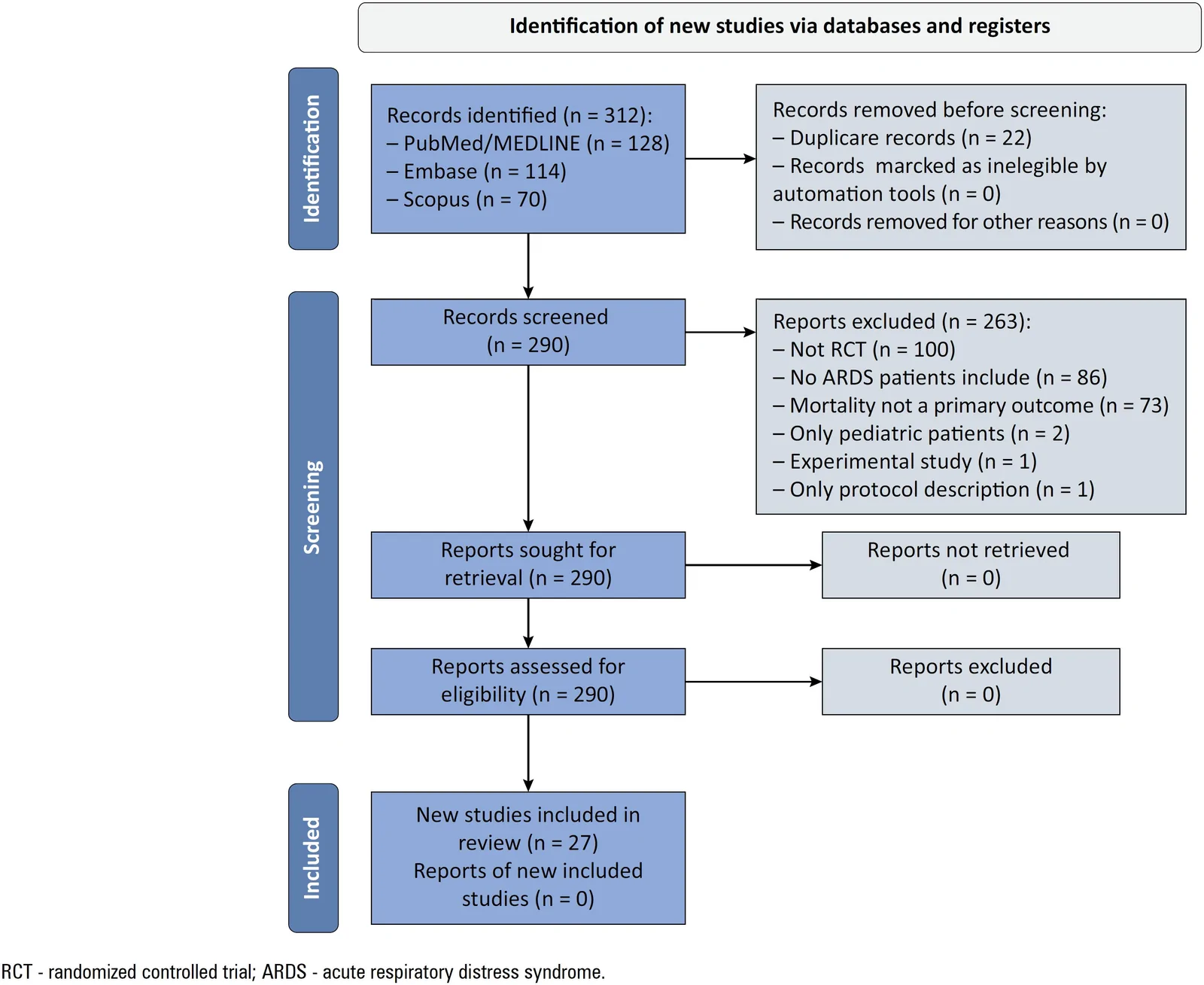
Study selection process.

A total of 312 records were identified through database searching, including 128 records from PubMed^®^/MEDLINE^®^, 114 from Embase, and 70 from Scopus. After removal of 22 duplicate records, 290 studies remained for screening. Following title and abstract screening, studies that were clearly unrelated to the study objective were excluded. Full-text assessment was subsequently performed for potentially eligible articles. A total of 263 studies were excluded after full-text review, mainly because they were not randomized controlled trials, did not include ARDS populations, did not evaluate mortality as a primary outcome, involved pediatric patients, or consisted only of study protocols. Ultimately, 27 randomized controlled trials met all eligibility criteria and were included in the final qualitative synthesis. The complete study selection process is presented in figure 1.

Tables 1 and 2 present the characteristics of the included randomized controlled trials and the representation of AKI-related variables across studies.

**Table 1:** Summary of randomized controlled trials investigating acute respiratory distress syndrome and mortality as primary outcome

Author	Study period	Sample size (n)	Center (n)	ARDS definition	ARDS severity	Intervention	Outcomes	COVID-19	Definition	Intervention Group	Control Group	Results
Bollen et al.^(12)^	October 1997 to March 2001	61	4 centers from United Kingdom, France, and Germany	PaO_2_/FiO_2_ < 200mmHg, radiographic evidence of bilateral infiltrates on CXR, and no evidence of atrial hypertension	Moderate to severe	High-frequency oscillatory ventilation	Primary: cumulative survival without MV or oxygen dependency at 30 days; mortality at 30 days; therapy failure; crossover rate; and persisting pulmonary problems defined as oxygen dependency or still being on a ventilator at 30 days	No	30 days	43%	33%	Neutral
Wiedemann et al.^(13)^	June 2000 to October 2005	1,000	20 centers from North America	PaO_2_/FiO_2_ < 300mmHg (adjusted if the altitude exceeded 1000m), and bilateral infiltrates on CXR consistent with the presence of pulmonary edema without evidence of left atrial hypertension	Mild, moderate, and severe	Conservative strategy of fluid management	Primary: death at 60 days Secondary: number of VFDs and organ-failure-free days and measures of lung physiology	No	60 days	25.5%	28.4%	Neutral
Wheeler et al.^(14)^	June 2000 to October 2005	999	20 centers from North America	Bilateral infiltrates on CXR consistent with the presence of pulmonary edema not due to left atrial hypertension, PaO_2_/FiO_2_ < 300mmHg	Mild, moderate, and severe	Pulmonary artery versus central venous catheter to guide treatment of acute lung injury	Primary: mortality during the first 60 days before discharge; Secondary: number of VFDs in the first 28 days; number of days without various types of organ failure	No	60 days	27.4%	26.3%	Neutral
Villar et al.^(15)^	March 1999 to March 2001		8 centers from Spain	ARDS American-European Consensus Conference criteria, PaO_2_/FiO_2_ ≤ 200mmHg	Moderate to severe	Pressure-controlled with spontaneous ventilation	Primary: ICU mortality Secondary: VFDs, pulmonary complications (barotrauma), extrapulmonary organ failures, and hospital mortality	No	ICU mortality	53.3%	32%	Neutral
Mercat et al.^(16)^	September 2002 to December 2005	767	37 ICUs from France	Recent appearance of bilateral pulmonary infiltrates consistent with edema, with no clinical evidence of left atrial hypertension; PaO_2_/FIO_2_ ≤ 300mmHg	Mild, moderate, and severe	Minimal distension strategy versus increased recruitment strategy	Primary: proportion of patients who died within 28 days after randomization Secondary: 60-day mortality, hospital mortality censored on day 60, numbers of VFDs and organ failure-free days from day 1 to day 28, and proportion of patients who experienced pneumothorax requiring chest tube drainage between day 1 and day 28	No	28 days	31.2%	27.8%	Neutral
Meade et al.^(17)^	August 2000 to March 2006	983	30 centers from Canada, Australia, and Saudi Arabia	New respiratory symptoms within 28 days and bilateral opacifications on CXR, PaO_2_/FIO_2_ ≤ 250 during invasive MV	Moderate to severe	Ventilatory strategy - combining low tidal volume, lung recruitment maneuvers, and high PEEP	Primary: all-cause hospital mortality Secondary: mortality during MV, ICU mortality, and 28-day mortality, and eligible use and total use of rescue therapies in response to refractory hypoxemia, refractory acidosis, or refractory barotrauma	No	Hospital mortality	36.4%	40.4%	Neutral
Taccone et al.^(18)^	February 2004 to June 2008	342	25 centers from Italy (23) and Spain (2)	ARDS American-European Consensus Conference criteria, PaO_2_/FiO_2_ ≤ 200mmHg	Moderate to severe	Prone positioning	Primary: 28-day all-cause mortality Secondary: 6-month mortality and mortality at ICU discharge, organ dysfunctions, and the complication rate related to prone positioning	No	28 days	31%	32.8%	Neutral
Papazian et al.^(19)^	March 2006 to March 2008	339	20 centers from France	PaO_2_/FiO_2_ ≤ 150mmHg with PEEP of 5 cm of water or higher and a tidal volume of 6 to 8mL per kg of predicted body weight, and bilateral pulmonary infiltrates that were consistent with edema	Severe	Cisatracurium continuous infusion	Primary: 90-day in-hospital mortality rate Secondary: day-28 mortality, numbers of days outside the ICU between day 1 and day 28 and between day 1 and day 90, number of days without organ or system failure between day 1 and day 28, rate of barotrauma, rate of ICU-acquired paresis, the MRC scores on day 28 and at the time of ICU discharge, and the numbers of VFDs between day 1 and day 28 and between day 1 and day 90	No	90 days	31.6%	40.7%	Positive
Spragg et al.^(20)^	November 2003 to March 2008	836	161 centers from 22 countries	PaO_2_/FiO_2_ < 170mmHg due to aspiration of gastric contents or pneumonia	Severe	Recombinant surfactant protein-C-based surfactant	Primary: percentage of patients alive at Day 28 Secondary: ventilator-free days at Day 28, measures of oxygenation and ventilation, and the number of nonpulmonary organ failure-free days	No	28 days	22.7%	23.8%	Negative
Gao Smith et al.^(21)^	December 2006 to March 2010	324	46 centers from United Kingdom	ARDS American-European Consensus criteria, PaO_2_/FiO_2_ ≤ 200mmHg	Moderate to severe	Intravenous salbutamol	Primary: 28-day mortality Secondary: mortality in the ICU or hospital before first discharge; ventilator-free and organ failure-free days from randomization to day 28, length of stay in ICU and hospital, and tachycardia, new arrhythmia, or other side-effects sufficient to stop treatment with trial drug	No	28 days	34%	23%	Negative
Young et al.^(22)^	December 2007 to July 2012	795	29 centers from England, Wales, and Scotland	PaO_2_/FiO_2_ ≤ 200mmHg with PEEP of 5cm of water or greater, if bilateral pulmonary infiltrates were visible on CXR without evidence of left atrial hypertension	Moderate to severe	High-frequency oscillatory ventilation	Primary: all-cause mortality 30 days after randomization; Secondary: all-cause mortality at the time of discharge from the ICU and the hospital, duration of MV, and use of antimicrobial, sedative, vasoactive, and neuromuscular-blocking drugs	No	30 days	41.7%	41.1%	Neutral
Guérin et al.^(23)^	January 2008 to July 2011	466	27 centers from France (26) and Spain (1)	ARDS American-European Consensus Conference criteria, PaO_2_/FiO_2_ < 150mmHg, with an FiO_2_ of ≥ 0.6, a PEEP of ≥ 5cm of water, and a tidal volume of about 6mL per kg of predicted body weight	Severe	Prone positioning	Primary: mortality at day 28 Secondary: mortality at day 90, rate of successful extubation, time to successful extubation, length of stay in ICU, complications, use of noninvasive ventilation, tracheotomy rate, number of days free from organ dysfunction, and ventilator settings, measurements of arterial blood gases, and respiratory-system mechanics during the first week after randomization	No	28 days	16%	32.8%	Positive
Truwit et al.^(24)^	March 2010 to September 2013	745	The National Heart, Lung, and Blood Institute Acute Respiratory Distress Syndrome and the Clinical Trials Network	Bilateral infiltrates on CXR that were consistent with pulmonary edema, without evidence of left atrial hypertension, PaO_2_/FiO_2_ ≤ 300mmHg.	Mild, moderate, and severe	Rosuvastatin	Primary: mortality before hospital discharge, home, or until study day 60 if the patient was still in a health care facility Secondary: VFDs to day 28, organ-failure-free days to day 14, ICU-free days to day 28, changes in the PaO_2_/FiO_2_ ratio and the oxygenation index during the first 7 days, hospital mortality to day 28, hospital-free days at day 60, VTE at day 14, composite end-point of myocardial infarction, bowel ischemia or ischemic stroke to day 28, arrhythmias during ICU stay or day 14, development of AKI, changes in plasma concentrations of CRP from day 0 to day 6 and 14	No	60 days	28.5%	24.9%	Negative
Willson et al.^(25)^	July 2008 to July 2011	427	34 centers from 6 countries	ARDS American-European Consensus Conference criteria, PaO_2_/FiO_2_ ≤ 200mmHg	Mild, moderate, and severe	Instilled calfactant	Primary: all-cause mortality at 90 days after study entry; Secondary: VFDs at 90 days, durations of ICU, hospital stay, and oxygen use, and changes in oxygenation after the study intervention	No	90 days	27.8%	25.5%	Negative
Cavalcanti et al.^(26)^	November 2011 to April 2017	1,010	120 centers from Brazil, Argentina, Colombia, Italy, Poland, Portugal, Malaysia, Spain, and Uruguay	ARDS American- European Consensus Conference criteria	Moderate to severe	Lung recruitment associated with PEEP titration according to the best respiratory system compliance	Primary: mortality until 28 days Secondary: length of ICU and hospital stay; VFDs from day 1 until day 28; pneumothorax requiring drainage within 7 days; barotrauma within 7 days; and ICU, in-hospital, and 6-month mortality	No	28 days	55.3%	49.3%	Negative
Combes et al.^(27)^	Until April 2017	249	60 centers from France, 1 from Australia, 2 from the USA and 1 from Canada	ARDS American-European Consensus Conference definition, PaO_2_/FiO_2_ < 50mmHg for > 3 hours, PaO_2_/FiO_2_ < 80mmHg for > 6 hours, or an arterial blood pH of < 7.25 with a PaCO_2_ of ≥ 60mmHg for > 6 hours, with the respiratory rate increased to 35 breaths per minute and MV settings adjusted to keep a plateau pressure of ≤ 32cm of water) despite ventilator optimization	Severe	ECMO	Primary: mortality at 60 days Secondary: treatment failure, mortality at other time points, the time to death until day 60, and a per-treatment analysis in which mortality was compared among patients who received ECMO and those who did not	No	60 days	35%	46%	Neutral
Moss et al.^(28)^	January 2016 to April 2018	1,006	48 centers from United States	PaO_2_/FiO_2_ ≤ 150mmHg with a PEEP of 8cm or more of water; bilateral pulmonary opacities on CXR or on computed tomography that could not be explained by effusions, pulmonary collapse, or nodules; and respiratory failure that could not be explained by cardiac failure or fluid overload	Moderate to severe	Cisatracurium continuous infusion	Primary: in-hospital death from any cause at 90 days Secondary: organ dysfunction (as assessed on the basis of the SOFA score, in-hospital death at day 28, days free of organ dysfunction, days not in the ICU, days free of MV, and days not in the hospital at day 28, long-term endpoints at 3, 6, and 12 months	No	90 days	35%	41.1%	Neutral
Beitler et al.^(29)^	October 2012 to September 2017	200	14 centers from United States and Canada	ARDS Berlin definition, PaO_2_/FiO_2_ ≤ 200mmHg	Moderate to severe	PEEP titration guided by esophageal pressure	Primary: ranked composite score that incorporated death and days free from MV through day 28 Secondary: all-cause mortality at 28 days, 60 days, and 1 year; VFDs through day 28; ICU and hospital lengths of stay through day 28 and day 60; protocol failure requiring rescue therapy; and functional status at 1 year	No	28 days	32.4%	30.6%	Neutral
Constantin et al.^(30)^	June 2014 to Feb 2017	400	20 centers from France	ARDS Berlin definition, PaO_2_/FiO_2_ ≤ 200mmHg	Moderate to severe	Personalized MV to lung morphology	Primary: all-cause mortality at day 90 Secondary: mortality at 28 days, 30 days, 180 days, and 365 days, ICU mortality, number of VFDs at day 30, ARDS resolution, length of stay in the ICU, the number of patients with VAP, and barotrauma	No	90 days	27%	27%	Neutral
Barrot et al.^(31)^	June 2016 to September 2018	201	13 centers from France	ARDS Berlin definition), PaO_2_/FiO_2_ ≤ 300mmHg	Mild, moderate, and severe	Conservative oxygen therapy (target PaO_2_, 55 to 70mmHg; oxygen saturation as measured by pulse oximetry [SpO_2_], 88 to 92%)	Primary: 28-day mortality Secondary: death in the ICU and at day 90; the SOFA scores (calculated without the respiratory component) at days 0, 3, and 7; VAP during the first 28 days; septicemia during the first 28 days; cardiovascular complications over the first 7 days; respiratory weaning success at days 28 and 90; RASS neurologic status, seizures, new cerebral stroke on imaging, administration of neuroleptics, and delirium	No	28 days	34.3%	26.5%	Neutral
Kumar et al.^(32)^	May 2020 to July 2020	30	4 centers from India	Moderate to severe ARDS; PaO_2_/FiO_2_ ratio of < 200 or more than 25% deterioration from the immediate previous value	Moderate to severe	Itolizumab	Primary: mortality 1 month after randomization, proportion of patients with deteriorating lung functions, proportion of patients who needed NIV, invasive MV/endotracheal intubation, and high flow nasal oxygen, and inflammatory markers: Ferritin, D-dimer, LDH, CRP Secondary: biomarkers such as IL-6, TNF-α, IL-17A, Absolute lymphocyte count, PaO_2_/FiO_2_ ratio, and number of participants with treatment-related side effects	Yes	30 days	0%	30%	Positive
He et al.^(33)^	November 2018 to September 2020	117	Single-center from China	ARDS Berlin definition, PaO_2_/FiO_2_ < 300mmHg	Mild, moderate, and severe	Electrical impedance tomography-guided PEEP	Primary: all-cause mortality within 28 days after randomization Secondary: number of VFDs at day 28, ICU length of stay, and new onset barotrauma during MV	No	28 days	21%	27%	Neutral
McNamee et al.^(34)^	May 2016 to December 2019	405	51 centers from United Kingdom	Moderate to severe hypoxemic respiratory; were receiving invasive MV using at least 5cmH_2_O of PEEP; PaO_2_/FiO_2_ < 150mmHg	Moderate to severe	Lower tidal volume ventilation facilitated by extracorporeal carbon dioxide removal	Primary: all-cause mortality 90 days after randomization; Secondary: tidal volume at day 2 and day 3, VFDs at day 28, duration of invasive MV in survivors, need for ECMO up to day 7, mortality at day 28, and adverse event rate	No	90 days	41.5%	39.5%	Neutral
Misset et al.^(35)^	September 2020 to March 2022	475	17 centers from Belgium	ARDS Berlin definition	Mild, moderate, and severe	Convalescent plasma	Primary: death by day 28 after randomization; Secondary: adverse events, inflammatory and anti-SARS-CoV-2 antibody responses, the SOFA score, the use of organ support, the length of hospital stay, and death by day 90 and 365	Yes	28 days	35.4%	45%	Positive
Richard et al.^(36)^	February 2013 to October 2018	699	22 centers from France	ARDS Berlin definition, PaO_2_/FiO_2_ ≤ 200mmHg	Moderate to severe	Pressure-controlled strategy allowing non-synchronized unassisted spontaneous ventilation (pressure-controlled with spontaneous ventilation)	Primary: in-hospital death from any cause at day 60 after randomization Secondary: mortality at day 28, numbers of VFDs, organ failure-free days, and vasoactive drugs-free days at day 28, total amounts of vasoactive drugs and fluids received during the first 7 days after inclusion, incidence of refractory hypoxemia during the first 7 days of treatment, proportion of patients requiring adjunctive treatment of hypoxemia during the first 7 days, number of days alive without sedation at day 28, total amounts of sedative drugs and NMBA during the first seven days after inclusion, mean sedation level from day 1 to day 7, duration of ventilation and ICU stay	No	60 days in hospital	34.6%	33.5%	Neutral
Tiwari et al.^(37)^	Not reported	30	Single center from India	Tested positive for Plasmodium falciparum, Plasmodium vivax, or both on a peripheral smear or rapid antigen test, with an acute onset of respiratory failure, bilateral infiltrates on CXR sparing the costophrenic angles, partial pressure of oxygen PaO_2_/FiO_2_ < 300mmHg, and lack of clinical evidence of left ventricular failure	Mild, moderate, and severe	Corticosteroid - pulse methylprednisolone therapy	Primary: death of the patient or discharge from the hospital Secondary: duration of stay in the medical ICU, the number of days for which MV was required, and the rate of secondary infections	No	Hospital mortality	40%	26.7%	Positive
Tongyoo et al.^(38)^	December 2010 to December 2014	197	Single center from Thailand	ARDS American-European Consensus definition, as later reclassified based on ARDS Berlin criteria	Mild, moderate, and severe	Hydrocortisone	Primary: all-cause mortality at study day 28 Secondary: patients alive without organ support (ventilator, renal replacement therapy, and vasopressors) on day 28, VFDs up to day 28, vasopressor-free days up to day 28, and mortality at study day 60	No	28 days	22.5%	27.3%	Neutral

ARDS - acute respiratory distress syndrome; PaO_2_/FiO_2_ - ratio of arterial oxygen partial pressure to inspired oxygen fraction; CXR - chest X-ray; MV - mechanical ventilation; VFD - ventilator-free day; ICU – intensive care unit; PEEP - positive end-expiratory pressure; VTE - venous thromboembolism; ECMO - extracorporeal membrane oxygenation; SOFA - Sequential Organ Failure Assessment; VAP - ventilator-associated pneumonia; SpO2 - peripheral capillary oxygen saturation; RASS - Richmond Agitation-Sedation Scale; LDH - lactate dehydrogenase; CRP - C-reactive protein; IL - interleukin; TNF-α - tumor necrosis factor-alpha; SARS-CoV-2 - Severe Acute Respiratory Syndrome Coronavirus 2; NMBA - neuromuscular blocking agent.

**Table 2:** Representation of acute kidney injury and renal outcomes in acute respiratory distress syndrome randomized controlled trials

Author	Study period	Sample size (n)	Creatinine levels	Coluna 1	Coluna 2	Coluna 3	AKI as a secondary outcome	AKI as an adverse event	AKI definition	AKI subgroup	RRT reported
Wiedemann et al.^(13)^	June 2000 to October 2005	1,000	No	Dependence on dialysis	NA	Yes	No	There were no significant differences between groups in the percentage of patients receiving kidney-replacement therapy (14% in the PAC group versus 11% in the CVC group, p = 0.15)	NA	No	Not reported
Meade et al.^(17)^	August 2000 to March 2006	983	No	No	NA	Yes	No	Not reported	NA	No	Not reported
Spragg et al.^(20)^	November 2003 to March 2008	836	No	No	NA	Yes	Organ failure-free days	Not reported	NA	No	Not reported
Truwit et al.^(24)^	March 2010 to September 2013	745	Yes	Dialysis requirement	Dialysis requirement	Yes	Number of organ failure-free days at 7 and 28 days after enrollment; dialysis to day 60	Not reported	Organ failure is defined as present on any date when the most abnormal vital signs/abnormal lab values meet the definition of clinically significant organ failure according to the Brussels organ failure table	No	Yes
Willson et al.^(25)^	July 2008 to July 2011	427	No	Other organ failure	NA	Yes	No	Renal failure	Renal failure	No	Not reported
Constantin et al.^(30)^	June 2014 to Feb 2017	400	No	No	Baseline 1% x 2%; RRT baseline 6% and 7%	Yes	No	Not reported	NA	No	Not reported
Misset et al.^(35)^	September 2020 to March 2022	475	No	No	NA	Yes	The number of organ failure-free days was defined as the number of days alive and free of organ failure	Not reported	Organ failures were defined using the Organ Dysfunctions and/or INfection score	No	Not reported

AKI - acute kidney injury; RRT - renal replacement therapy; NA - not available; PAC - pulmonary artery catheter; CVC - central venous catheter.

### Study characteristicst

Of the 27 included studies, 24 were conducted as multicenter trials,^(12-32,34-36)^ and 3^(33,37,38)^ were single-center. Two studies enrolled patients exclusively with COVID-19 related ARDS,^(32,35)^ whereas 25 studies investigated ARDS of other etiologies. The classification of ARDS varied among the included studies. A total of 17 studies specifically enrolled patients with moderate and severe ARDS. Ten studies defined ARDS based on partial pressure of oxygen/fraction of inspired oxygen (PaO_2_/FiO_2_), thereby potentially including patients across the mild, moderate, and severe spectrum.^(13,14,16,24,25,31,33,35,37,38)^ and only four studies explicitly focused on severe ARDS.^(19,20,23,27)^

The included randomized controlled trials investigated a wide range of interventions for ARDS. Ventilatory strategies were the most frequently studied, appearing in 12 trials.^(12,15,16,17,22,26,29,30,31,34,36)^ Other commonly evaluated interventions included neuromuscular blockade (two trials),^(19,28)^ prone positioning (two trials),^(18,23)^ and corticosteroids (two trials).^(37,38)^ All pharmacologic and supportive strategies are detailed in table 1.

### Reporting of acute kidney injury and related renal variables

Acute kidney injury was infrequently specified as a standalone secondary outcome. Most studies reported kidney-related outcomes indirectly, embedded within composite measures such as organ failure-free days, Sequential Organ Failure Assessment (SOFA) scores, RRT-free days, or duration of RRT. Table 2 summarizes how AKI and related renal variables were reported across included trials. Only seven studies reported AKI or any kidney outcome as an adverse event. No study employed formal AKI definitions, such as those proposed by KDIGO, AKIN, or RIFLE. Renal dysfunction, when reported, was variably defined using RRT use, serum creatinine thresholds, or global organ failure frameworks Organ Dysfunctions and/or INfection (ODIN) score or Brussels Organ Failure Table). Serum creatinine values were reported in two studies,^(24,28)^ either at baseline or during the follow-up period.

Renal replacement therapy use was reported in four studies.^(15,16,24,27)^ In these, RRT was presented as the proportion of patients receiving therapy in each arm, without additional details regarding modality, timing, or criteria for initiation. No study reported the proportion of patients with AKI at enrollment or the incidence of AKI during the study follow-up. Similarly, none of the included studies reported a specific mortality analysis for the AKI subgroup.

### Reporting of fluid balance

Fluid balance data were available in five studies, as shown in table 2.^(17,20,24,30,35)^ In most cases, fluid balance was reported at early time points, such as within 24 to 48 hours after randomization. None of the included studies incorporated fluid balance into adjusted analyses or explored its association with mortality or organ dysfunction.

### Mortality outcomes

The mortality endpoint was consistently reported across all included studies, although with variable time frames, as detailed in table 1. Most trials reported short- to mid-term mortality, with 28-day mortality being the most common endpoint,^(16, 18,20,21,23,26,29,31,33,35,38)^ followed by 90-day^(19,25,28,30,34)^ and 60-day mortality,^(13,14,24,27)^ while a minority used hospital mortality.^(17,37)^ This variability in mortality assessment was accompanied by the absence of predefined mortality analyses stratified by kidney-related variables, including AKI status, across the included trials.

## DISCUSSION

This systematic review highlights a persistent and clinically relevant omission in the design of RCTs in ARDS. Although AKI is a frequent complication in moderate to severe ARDS and independently contributes to worse outcomes, kidney-specific endpoints were rarely incorporated. Most trials did not apply standardized AKI definitions, such as KDIGO, and reporting of renal outcomes, including serum creatinine trajectories or use of RRT, was inconsistent or absent. This lack of structured renal assessment limits the interpretability of multiorgan effects and hinders efforts to advance organ-supportive strategies in this population.

Importantly, part of this heterogeneity must be interpreted in light of the historical evolution of AKI definitions. The RIFLE criteria were first proposed in 2004, followed by the AKIN classification in 2007, while the KDIGO consensus - currently the most widely adopted framework - was published in 2012 and progressively incorporated into ICU research thereafter.^(39)^ Consequently, many earlier ARDS trials could not have applied KDIGO-based definitions, and even post-2012 studies showed variable uptake of standardized criteria. While this temporal context may clarify some variability in reported renal outcomes across trials, it does not fully explain the continued lack of strict renal endpoints in recent studies.

Indeed, none of the included trials employed standardized AKI definitions such as KDIGO, despite their broad acceptance and incorporation into ICU research since 2012.^(39,40)^ Instead, several studies referenced the SOFA score but failed to clarify whether and how the renal subscore was used. This ambiguity limits the clinical interpretability of renal dysfunction and precludes valid cross-trial comparisons.^(40)^ Furthermore, reporting of serum creatinine and RRT was often incomplete, lacking key clinical details such as timing, modality, and initiation thresholds. These methodological gaps are particularly concerning given the well-established prognostic value of renal function and RRT parameters in critically ill populations.^(41,42)^

These findings should be interpreted within the broader context of critical care research, in which randomized trials have historically emphasized all-cause mortality as the principal marker of therapeutic benefit.^(43)^ In syndromes characterized by complex and interacting pathophysiological processes, such as ARDS, interventions may exert heterogeneous or modest effects on survival, while substantially influencing other domains of organ dysfunction. The limited and inconsistent reporting of renal variables observed across the included trials likely reflects this historical prioritization of mortality, rather than an absence of renal involvement or clinical relevance. Our results suggest that, within this framework, kidney-related outcomes have not been systematically incorporated, even when AKI represents a frequent and prognostically relevant complication.

In parallel, growing recognition of ARDS as a biologically heterogeneous syndrome, together with ongoing efforts to refine ARDS definitions and improve patient classification, provides an important interpretative lens for our findings.^(44)^ Distinct subphenotypes with differing inflammatory profiles and degrees of extrapulmonary organ dysfunction have been associated with divergent clinical trajectories and outcomes.^(45)^ Our review indicates that this biological heterogeneity has rarely been accounted for in ARDS trials, as reflected by the absence of stratified analyses or structured assessment of kidney dysfunction. The limited characterization of extrapulmonary organ involvement may therefore contribute to incomplete interpretation of trial results and missed signals of differential treatment effects.^(30)^

Fluid balance - a key determinant of both pulmonary and renal outcomes in critical illness - was infrequently reported. Fewer than 20% of trials included data on cumulative fluid status, and only a minority incorporated it into adjusted analyses. This omission is particularly notable given evidence from the FACTT trial and subsequent cohort studies demonstrating fluid overload contributes to both pulmonary edema and AKI.^(13,46)^ Failure to account for fluid dynamics may obscure the renal consequences of lung-targeted interventions and limit the detection of potential harm in susceptible subgroups.^(6)^

None of the included trials conducted stratified analyses by AKI status or examined potential heterogeneity in treatment effects by baseline kidney function.^(47)^ This represents a missed opportunity to evaluate whether patients with impaired renal reserve respond differently to interventions in ARDS. Renal dysfunction is known to alter drug pharmacokinetics, modulate systemic inflammation, and influence hemodynamic responses.^(48)^ These alterations may interact with fluid balance - a key determinant of both pulmonary and renal outcomes - as well as with therapies such as lung-protective ventilation, corticosteroids, and extracorporeal modalities.^(46)^ Moreover, mechanical ventilation itself - particularly when associated with ventilator-induced lung injury - can propagate kidney injury through biotrauma and altered hemodynamics.^(48)^ Identifying such cross-organ interactions is essential to advancing precision medicine and should be prioritized in future trial designs.^(47,49)^ Furthermore, recent advances in AKI subphenotyping and personalized medicine may help identify biologically distinct patient groups that respond differently to therapeutic interventions, thereby improving future ARDS trial design and interpretation.^(50)^ The broader issue reflected in these findings is the persistence of a single-organ approach in critical care trials. ARDS continues to be framed primarily as a pulmonary condition, despite growing recognition of its systemic implications. Lung-kidney crosstalk is well established and involves mechanical, inflammatory, and neurohormonal pathways. By neglecting kidney-specific endpoints, current RCTs risk overlooking meaningful effects or unintended harms, especially in patients with overlapping vulnerabilities.^(5,6)^

Renal replacement therapy, a high-cost, high-stakes intervention, was rarely analyzed despite being documented in several trials. Without reporting RRT timing or stratifying mortality by its use, trial conclusions may mask differences in severity or misattribute outcomes. This omission weakens both internal validity and external applicability, particularly for ICU clinicians managing patients with evolving multiorgan failure.^(51,52)^

### Strengths and limitation

This review has several strengths. It is, to our knowledge, the first to systematically assess how AKI is reported in RCTs of ARDS with mortality endpoints. The review included trials identified from three major biomedical databases and covered more than two decades of randomized evidence in ARDS. By restricting the analysis to trials in which mortality was a central outcome, we focused on studies that have disproportionately influenced evidence synthesis and clinical practice in this field.

However, some limitations must be acknowledged. We limited inclusion to English-language studies, which may have excluded relevant international trials. We did not perform a formal risk-of-bias assessment, as the review was descriptive and not focused on treatment efficacy. Additionally, our analysis relied on reported data; some studies may have collected renal variables but chose not to report them due to space constraints or prioritization of respiratory endpoints. Finally, heterogeneity in ARDS and AKI definitions over time likely contributed to variability in study populations and outcome reporting across included studies.

## CONCLUSION

Acute kidney injury is a frequent and clinically meaningful complication in acute respiratory distress syndrome, yet it remains inconsistently represented in randomized trials evaluating mortality. This pattern of reporting limits the interpretability of trial findings regarding extrapulmonary organ dysfunction, particularly in patients at higher risk of renal involvement. Future acute respiratory distress syndrome trials would benefit from the use of standardized acute kidney injury definitions, more consistent reporting of renal replacement therapy and fluid balance, and consideration of renal function in exploratory or stratified analyses. Recognizing the multisystem nature of critical illness is essential to designing trials that are both scientifically sound and clinically useful.

## Data Availability

All data generated or analyzed during this study are included in this published article.
